# Multiple Minute Gallbladder Muscle Calcifications

**DOI:** 10.1055/s-0041-1731449

**Published:** 2021-07-22

**Authors:** Adriana Handra-Luca

**Affiliations:** 1 Service d'Anatomie Pathologique, APHP GHU Avicenne, Bobigny, France; 2 University Sorbonne Paris Nord, UFR SMBH, Bobigny France

**Fig. 1:**
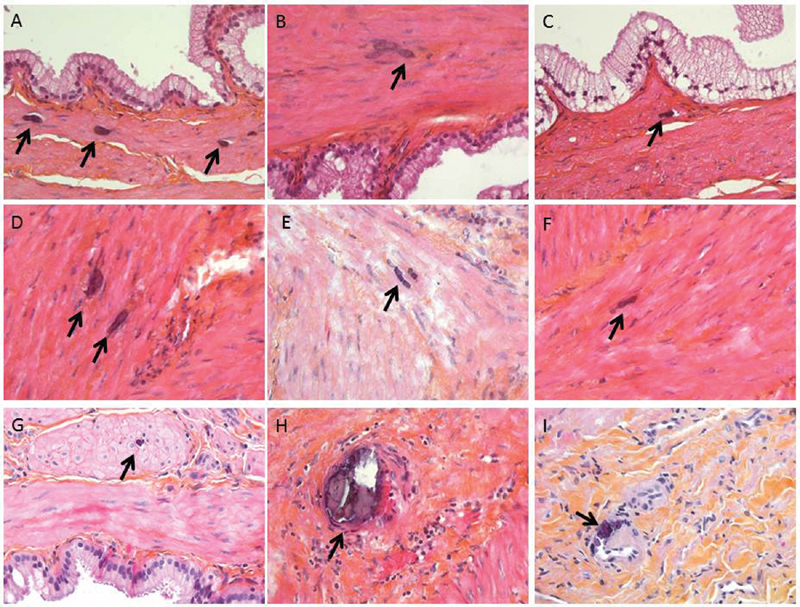
The calcifications were located at direct contact to the gallbladder muscle (A–F). Some of them were also at contact to the inter-muscle cell connective tissue. Calcifications were also observed at contact to capillary vessel wall (in the muscle layer). To note would be the presence of granular dystrophia of muscle cells (G). Original magnification ×40 (A–I).

We have read with great interest the article of Iqbal et al reporting cases of porcelain gallbladder. 1 Most reported cases show calcifications when at an extensive stage or of bone metaplasia type. 2 3 However, other types of calcifications can be detected in the gallbladder wall, for example, intracellular epithelial mucosal calcifications in chronic cholecystitis. 4 We would like to draw attention on another type of gallbladder calcifications that of multiple, minute, muscle layer calcifications ( Fig. 1 ). Most calcifications were situated between the muscle cells or between muscle cells and the connective tissue (fibrotic or not) of the muscle layer. Some calcifications were situated in zone of muscle granular degeneration/dystrophia and at contact to capillary walls (without calcifications in arteries or veins). The number of reactive lymphocytes was not increased at contact. The gallbladder wall showed mild subacute and chronic cholecystitis.

The histogenesis of this type of extraskeletal calcification is difficult to precise. Renal failure might be incriminated since the medical history revealed increased serum creatinine, cortical right renal cyst in the context of type-2 diabetes diagnosed 5 years previously. Calcifications were detected in the aortic valves, abdominal aorta, iliac and femoral and popliteal arteries, and microcalcifications in the liver (segments 5, 7, and 8), while bone demineralization was diagnosed on computed tomography (CT)-scan. An additive/favoring effect of drugs taken cannot be ruled out, ramipril/hydrochlorothiazide being known to raise serum creatinine, uric acid, as well as calcium and levetiracetam/parahydroxybenzoate/maltitol, renal failure. 5 6 Moreover, muscle degeneration was detected at contact of some of the calcifications, possibly result of ischemia as related to the cirrhosis-related vascular changes and to atorvastatine-related muscle abnormalities. 7

In conclusion, we report multiple, minute gallbladder calcification located in the muscle layer, perivasculary or not. Such lesions of complex etiology and possibly corresponding to an incipient stage of porcelain gallbladder might be of potential clinical relevance for imaging diagnosis.
